# Early immune events during acute HIV infection

**DOI:** 10.1186/1742-4690-9-S2-P181

**Published:** 2012-09-13

**Authors:** BM Slike, B Tassaneetrithep, S Nitayaphan, K Rono, E Sanga, A Sekiziyivu, D Stablein, L Eller, MA Eller, J Kim, N Michael, M Robb, M Marovich

**Affiliations:** 1 Siriraj Hospital, Mahidol University, Bankok, Thailand; 2 Armed Forces Research Institute of Medical Sciences, Bankok, Thailand; 3 Walter Reed Program, Kericho, Kenya; 4 Mbeya Medical Research Programme, Mbeya, Tanzania, United Republic of; 5 Makerere University Walter Reed Project, Kampala, Uganda; 6 Emmes Corporation, Rockville, MD, USA; 7 US Military HIV Research Program, Silver Spring, MD, USA

## Background

We characterized plasma cytokine and chemokine profiles at baseline and during acute infection in a prospective high-risk cohort (RV217). PBMC collected in parallel allow for the longitudinal analysis of changes in immune cell populations during acute infection. These data can provide insight into the earliest events in host-HIV interaction and inform HIV vaccine development.

## Methods

Semiweekly viral load (VL) testing in individuals at high-risk for HIV acquisition was performed to prospectively identify very early acute HIV infections (Fiebig stage I/II, NAT+/Ab-). Cytokines were assayed in plasma from pre-infection through early plasma viral load set point using the Q-Plex Multiplex Array or by traditional ELISA. Peripheral blood cells were longitudinally analyzed with multiparameter flow cytometry.

## Results

African participants were all female and acquired subtype A, C and D recombinant HIV while participants in Thailand were male and acquired HIV subtype E predominantly. Longitudinal plasma samples from acutely infected individuals (n=24) showed similar trends in cytokine profiles with statistically significant increases in expression over time. VL setpoint, IL-10 and MCP-1 expression differed by region. Preliminary PBMC analysis revealed frequency and phenotypic changes in all antigen presenting cell populations. Significant decreases in plasmacytoid dendritic cells were observed at peak VL, which preceded a sharp decline in plasma IFN-α to near baseline values. MCP-1 and IP-10 also rose sharply in conjunction with fluctuations in T cell subsets and APCs. These early changes could impact adaptive cellular and humoral immune responses.

## Conclusion

Understanding cytokine kinetics and VL dynamics during acute HIV infection may help identify key controls of early viral set point. Regional differences may reflect HIV subtype, gender, background cytokine expression levels and co-morbidities as these covaried. Analyses of correlations with immune cell phenotype/function and viral set point are underway. Efforts focus on examination of the evolution of the innate response after infection, prior to peak viremia.

